# Codelivery of TGF-β1 and anti-miR-141 by PLGA microspheres inhibits progression of intervertebral disc degeneration

**DOI:** 10.1186/s13018-023-03501-5

**Published:** 2023-01-06

**Authors:** Liang Xiao, Daokuan Gao, Yu Zhang, Chen Liu, Zongsheng Yin

**Affiliations:** 1 Department of Orthopaedics, The First Affiliated Hospital of Anhui Medical University, Hefei, 230022 China; 2 Department of Spine Surgery, Yijishan Hospital, The First Affiliated Hospital of Wannan Medical College, Wuhu, 241001 China; 3 Department of Orthopaedics, The First Affiliated Hospital of Anhui Medical University, 218 JiXi Road, Hefei, 230022 Anhui China

**Keywords:** Microsphere, TGF-β1, miR-141, Nucleus pulposus, Intervertebral disc degeneration

## Abstract

**Background:**

Cervical and lumbar pain is usually caused by degeneration of the nucleus pulposus (NP). As a powerful therapeutic strategy, tissue engineering can effectively restore the normal biological properties of the spinal unit. Previous studies suggested that poly(lactic-co-glycolic acid) (PLGA) microspheres are effective carriers of cells and biomolecules in NP tissue engineering. This study aims to explore the therapeutic effect of PLGA microspheres coloaded with transforming growth factor-β1 (TGF-β1) and anti-miR-141 on intervertebral disc degeneration (IDD).

**Methods:**

PLGA microspheres were characterized by scanning electron microscopy, a laser particle size analyzer, and laser confocal microscopy. The in vitro release rate of biomolecules from the microspheres was analyzed by reversed-phase high-performance liquid chromatography and agarose gel electrophoresis. The rat NP cells (NPCs) treated with the solutions released from microspheres for different lengths of time were assigned to a control group (Ctrl), an empty PLGA microsphere group (Mock microsphere, MS), a TGF-β1-loaded PLGA microsphere group (TMS), an anti-miR-141-loaded PLGA microsphere group (AMS), and an anti-miR-141 + TGF-β1-loaded PLGA microsphere group (ATMS). The proliferation and apoptosis of NPCs were observed by alamar blue and flow cytometry. The gene and protein expression of cartilage markers COL2A1 and ACAN were observed by RT-qPCR and Western blot. The rat model of IDD was established by tail puncture. Rats were divided into a control group (Ctrl), a mock operation group (Mock), a TGF-β1 microsphere group (TMS), an anti-miR-141 microsphere group (AMS), and an anti-miR-141 + TGF-β1 microsphere group (ATMS). The degree of rat tail IDD was assessed in each group through magnetic resonance imaging (MRI), safranin O-fast green staining, immunohistochemistry, and Western blotting.

**Results:**

PLGA microspheres were stably coloaded and could sustainably release TGF-β1 and anti-miR-141. The results of in vitro cell experiments showed that the release solution of PLGA microspheres significantly enhanced the proliferation of NPCs without inducing their apoptosis and significantly upregulated cartilage markers in NPCs. The effect of microspheres was greater in the ATMS group than that in the TMS group and AMS group. In vivo experiments showed that IDD could be effectively inhibited and reversed by adding microspheres coloaded with TGF-β1 and/or anti-miR-141, and the effect was greatest in the ATMS group.

**Conclusion:**

PLGA microspheres coloaded with TGF-β1 and anti-miR-141 can reverse IDD by inhibiting the degeneration of NPCs.

## Introduction

Cervical and lumbar pain, a common orthopedic disease, seriously affects the work and life of patients, and intervertebral disc degeneration (IDD) is considered the initiating factor for the occurrence and progression of cervical and lumbar pain [1, 2]. The intervertebral disc is composed of the annulus fibrosus, nucleus pulposus (NP), and cartilage endplates. NP degeneration is thought to be the earliest change of IDD as well as the most critical step in the IDD process [3, 4]. The NP can keep its homeostatic balance under normal circumstances. In the case of NP degeneration, such homeostasis is broken, thus causing pathological changes that mainly manifest as a decrease in NP cells (NPCs), loss of proteoglycan in the extracellular matrix, and disorders of collagen structure. Ultimately, dehydration, revascularization, and re-neuralization of NP occur, leading to loss of function and causing corresponding clinical symptoms [5, 6]. Therefore, exploring the degeneration and regenerative repair of NP tissues is the core of the IDD research field [7].

Due to the poor self-repair ability of NP tissues, IDD is rarely self-reversable. An ideal therapy could be to promote the secretion of extracellular matrix by NPCs and inhibit relevant catabolic enzymes through the actions of biological agents [8]. Transforming growth factor-β1 (TGF-β1) has a key regulatory role in IDD [9, 10]. Injecting TGF-β1 into the degenerated intervertebral disc can exert a definite repair effect on the intervertebral disc [11]. The in vivo use of TGF-β1 is restricted due to its easy degradation, short half-life, and difficulty of long-term stable expression [12].

miRNAs, a class of endogenous, non-coding, small RNA molecules, can regulate the stability and transcriptional expression of mRNAs. Abnormally expressed miRNAs are important regulators in the pathophysiological process of IDD [13]. Applying miRNAs to the repair in the field of regenerative medicine is becoming a new research hotspot [14–18]. However, miRNAs are also prone to degradation in vivo, so carriers are required for their efficient and safe transfection.

In recent years, growing attention has been paid to microparticle systems in tissue engineering. With excellent biocompatibility and a more uniform degradation rate, poly(lactic-co-glycolic acid) (PLGA) can meet different needs of tissue engineering if its composition ratio and molecular weight are adjusted [19]. In this study, injectable microspheres were constructed based on PLGA, and TGF-β1/anti-miR-141 complexes were loaded on PLGA microspheres for in situ delivery. The microspheres significantly inhibited the pathological process of NP degeneration and exerted a definite therapeutic effect on IDD. This study offers a new strategy for exploring the biological repair of IDD.

## Materials and methods

### Preparation of PLGA microspheres coloaded with TGF-β1 and anti-miR-141

PLGA microspheres coloaded with TGF-β1 and anti-miR-141 were prepared by the water-in-oil emulsification/solvent evaporation method [20]. First, 150 mg of PLGA was dissolved in 2 mL of dichloromethane and added to 0.2 mL of 0.5 mg TGF-β1 or anti-miR-141. The mixture was put in an ice bath and emulsified using a homogenizer (8000 rpm, 30 s). Then 0.1 mL of ammonium bicarbonate solution (100 mg/mL) was added to the system. The mixture was further emulsified using the homogenizer (4000 rpm, 30 s), and the emulsion was then added to 50 mL of polyvinyl alcohol solution, followed by homogenization (4000 rpm, 2 min). The resulting product was added to 50 mL of deionized water and magnetically stirred for more than 3 h to volatilize dichloromethane. Finally, after centrifugation (8000 rpm, 10 min), the PLGA microspheres were collected, washed with deionized water three times to remove the free non-sphered components, freeze-dried, and stored at − 20 °C for later use. The empty PLGA microspheres (Mock microsphere, MS), PLGA microspheres loaded with TGF-β1 (TMS) or anti-miR-141 alone (AMS), and PLGA microspheres coloaded with both TGF-β1 and anti-miR-141 (ATMS) were prepared in the above way.

### Characterization of PLGA microspheres

The surface morphological characteristics of PLGA microspheres were observed by scanning electron microscopy under an accelerating voltage of 5 kV. The particle size of PLGA microsphere was measured using a laser particle size analyzer. To determine the distribution of TGF-β1 and anti-miR-141 in the microspheres, fluorescein-labeled PLGA microspheres were prepared and observed by laser confocal microscopy.

### In vitro biomolecule release assay

The release status of TGF-β1 and anti-miR-141 from PLGA microspheres was detected at pH 7.4. First, PLGA microspheres were placed in a dialysis cassette and immersed phosphate-buffered saline (PBS) at pH 7.4 in a large beaker. A certain volume of sample was collected from the external solution chamber at predetermined intervals, and the PBS was replenished with the same volume of pH-adjusted fresh water. The level of TGF-β1 in the sample was detected by reversed-phase high-performance liquid chromatography. The presence or absence of anti-miR-141 in the sample was detected by agarose gel electrophoresis and quantified using a Quant-It™ PicoGreen dsDNA kit.

### Cell proliferation assay

The spine-derived NPCs of Sprague–Dawley rats were plated in a 96-well plate (9.0 × 10^3^/well) and cultured with Dulbecco’s modified Eagle’s medium (DMEM) in a 5% CO_2_ incubator at 37 °C for 24 h. Twenty microliters of 5-, 10-, 20-, and 40-d PLGA microsphere release solution were added for 24-h treatment. Then the NPCs were incubated with 20 µL of DMEM medium containing 10% Alamar blue and 10% fetal bovine serum for 2 h, and the medium was aspirated (100 µL/well). The absorbance values at 570 nm and 600 nm were measured with a microplate reader. The corresponding sample without cells was inoculated as a blank control in each group. Finally, the relative cell count was determined: cell proliferation rate = absorbance_experimental group_/absorbance_control group_.

### Apoptosis assay

The NPCs were plated in a six-well plate (2.5 × 10^5^/well) containing 2 mL of DMEM and incubated at 37 °C overnight. After the medium was discarded, 2 mL of medium containing 100 µL of 15-d microsphere release solution was added. Twenty-four hours later, the NPCs were digested with trypsin, centrifuged, and washed twice with PBS. Then they were resuspended with binding buffer according to the kit instructions and incubated with Annexin V-FITC and PI at room temperature away from light for 10 min. Finally, apoptosis was detected by a flow cytometer (BD Biosciences, Mountain View, USA).

### RT-qPCR

Total RNA was extracted from cells by TRIzol (Invitrogen, USA). The ACAN and COL2A1 mRNA expression level was measured using the SYBR® Premix Ex Taq™ II kit (Takara, Japan). The PCR program was as follows: pre-denaturation at 95 °C for 30 s, and 50 cycles of denaturation at 95 °C for 5 s and annealing/extension at 60 °C for 25 s. Relative quantification was performed by the 2^−△△Ct^ method, with GAPDH as an internal control. The assay was repeated three times. The primer sequences are shown in Table 1.

**Table 1:** Primers and sequences used in this study

Gene	Primer	Primer sequence (5′ → 3′)
ACAN	F	CATTCACCAGTGAGGACCTCGT
ACAN	R	TCACACTGCTCATAGCCTGCTTC
COL2A1	F	TGAGGGCGCGGTAGAGACCC
COL2A1	R	TGCACACAGCTGCCAGCCTC
GAPDH	F	GCTGAGAACGGGAAGCTTGT
GAPDH	R	GACTCCACGACGTACTCAGC

### Western blotting

The cell or tissue samples were collected, washed twice with PBS, and lysed with radioimmunoprecipitation assay lysis buffer for 2 h on ice, with protease inhibitors added. The lysate was centrifuged at 12,000 rpm for 10 min, and the supernatant was harvested. The proteins were quantified using a bicinchoninic acid kit. Then an equal amount of protein was separated by sodium dodecyl sulfate–polyacrylamide gel electrophoresis, and the protein of a specific molecular weight was electro-transferred onto a polyvinylidine fluoride membrane. The membrane was blocked with PBS containing 5% skim milk powder and 1% Tween-20 (PBST) at room temperature for 1 h, then incubated with primary antibodies (ACAN, COL2A1) at 4 °C overnight. After washing three times with PBST, the membrane was incubated with horseradish peroxidase–labeled secondary antibodies at room temperature for 1 h. Finally, specific protein bands were detected by chemiluminescence solution.

### Immunofluorescence assay

The NPCs were fixed with 4% paraformaldehyde, permeabilized with 0.3% Triton X-100, and blocked with 2% sheep serum, followed by incubation with primary antibodies (ACAN, COL2A1) (1:1000, Abcam, USA) at 4 °C overnight. Following incubation with fluorescent secondary antibodies and DAPI, the NPCs were washed with PBS, and the change in the target protein content was observed under a laser confocal microscope (Leica, Germany).

### Animal experiments

Upon the approval by the Laboratory Animal Committee of the support unit, 40 8-week-old Sprague–Dawley rats were randomly divided into a control group (Ctrl), an operation group (Mock), a TGF-β1 microsphere group (TMS), an anti-miR-141 microsphere group (AMS), and an anti-miR-141 + TGF-β1 microsphere group (ATMS), with eight rats in each group. No operation was performed in the Ctrl group. In the Mock, TMS, AMS, and ATMS groups, the rats were anesthetized with pentobarbital (40–50 mg/kg) and fixed in a supine position. After hair shaving, the abdomen was cleaned, disinfected with iodophor, and draped. Then the rat tail intervertebral disc was punctured till the central NP with a 21-G micropuncture needle (Hamilton, Bonaduz, Switzerland) under X-ray fluoroscopy. The needle was rotated 360° and then withdrawn. The puncture site was sterilized with iodophor, and 50,000 U of penicillin was injected intramuscularly to prevent infection. In the TMS, AMS, and ATMS groups, the rats were additionally injected with TMS, AMS, and ATMS in the operative intervertebral space by a 33G micropuncture needle, after which the puncture site was also sterilized with iodophor and 50,000 U of penicillin was injected intramuscularly to prevent infection. The IDD status was observed by lumbar MRI plain scan at 2 months after modeling and was evaluated by Pfirrmann grading in each group. After all rats were sacrificed, the tail intervertebral disc samples were harvested. The morphological changes in the operative segment of intervertebral disc were observed by safranin O-fast green staining. The expression of COL2A1 in the NP tissues of the operative segment was detected by immunohistochemistry and Western blotting. The intervertebral disc was stained by histologically, and the results were graded and compared according to the criteria of Masuda et al. [21].

### Statistical analysis

SPSS18.0 software was used for statistical analysis. Normally distributed measurement data are described as mean ± standard deviation. The data were compared for significance using the independent-samples *t*-test between two groups and using one-way analysis of variance between more than two groups. Before the analysis of variance, the homogeneity test of variance was routinely performed. Tukey’s highly significant difference test was used to compare the significance of differences in the case of homogeneity of variances, while Dunnett’s T3 test was used in the case of heterogeneity of variances. *P* < 0.05 was considered statistically significant.

## Results

### Characterization of PLGA microspheres and the loading and release of biomolecules

**Fig. 1:**
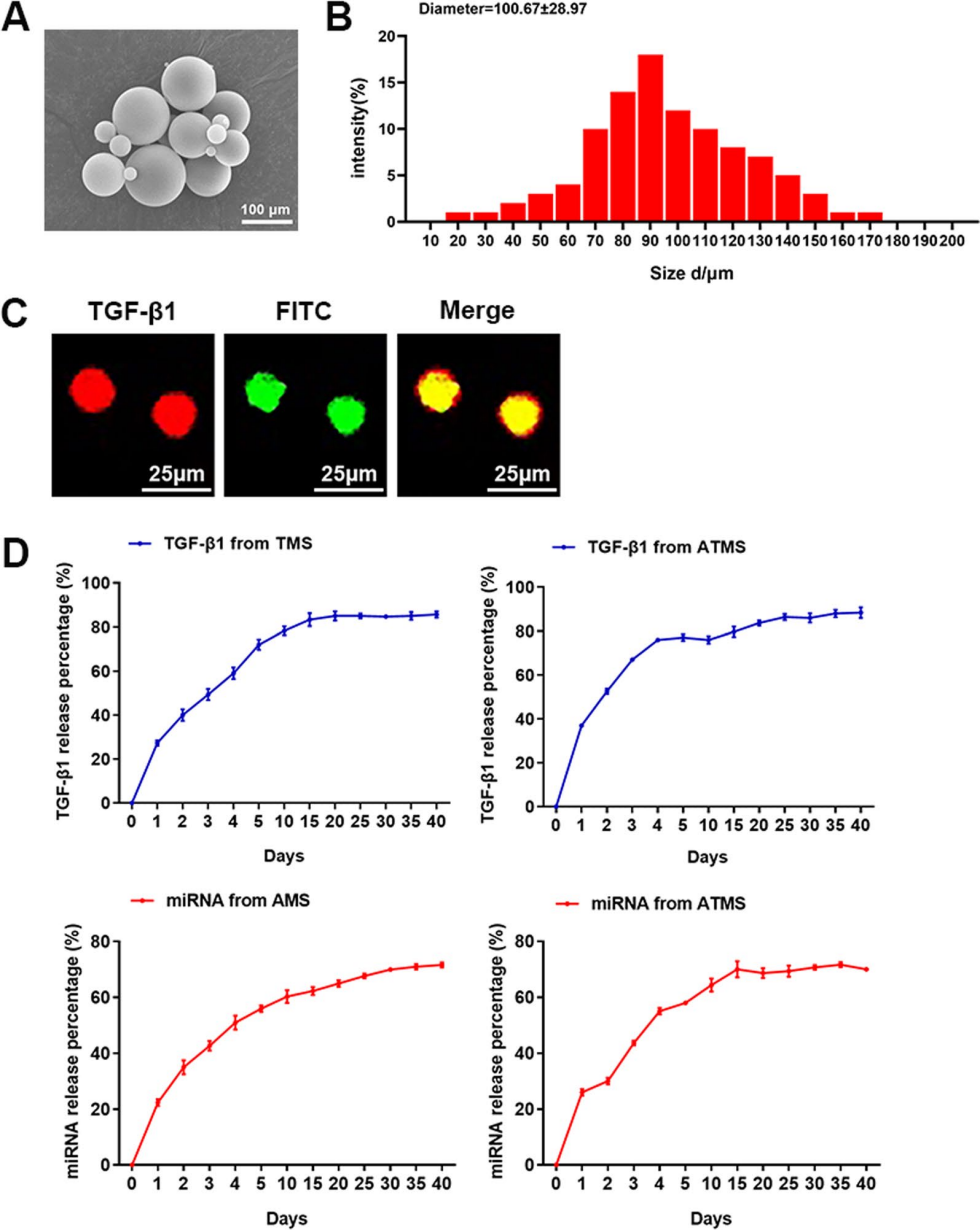
Characterization of PLGA microspheres and the loading and release of biomolecules. **A**, **B** SEM images of PLGA microsphere. **C** Localization of TGF-β1 and anti-miR-141 in microsphere. **D** Release detection of TGF-β1 and anti-miR-141 in vitro

Scanning electron microscopy showed that the PLGA microspheres had a uniform spherical shape and did not adhere to each other. The mean particle size was 100 µm (20–170 µm) (Fig. 1A, B). To directly verify the coloading of TGF-β1 and anti-miR-141 in PLGA microspheres, red TGF-β1 fluorescence and green FITC fluorescence (FITC-labeled anti-miR-141) in PLGA microspheres were observed by laser confocal microscopy. In the overlap images, PLGA microspheres displayed yellow fluorescence, suggesting that both TGF-β1 and anti-miR-141 were uniformly dispersed in the microspheres (Fig. 1C). To determine the release efficiency of TGF-β1 and anti-miR-141 from PLGA microspheres, the release solution of single-loaded and coloaded PLGA microspheres was detected for 40 consecutive days. As shown in Fig. 1D, both TGF-β1 and anti-miR-141 showed a slow release trend, and the biomolecule release rate was roughly the same between the single-loaded and coloaded PLGA microspheres. The release efficiency of TGF-β1 and anti-miR-141 reached a plateau at approximately 15 d, so the 15-d release solution of PLGA microspheres was selected for later experiments.

### Effect of PLGA microsphere release solution on NPCs

**Fig. 2:**
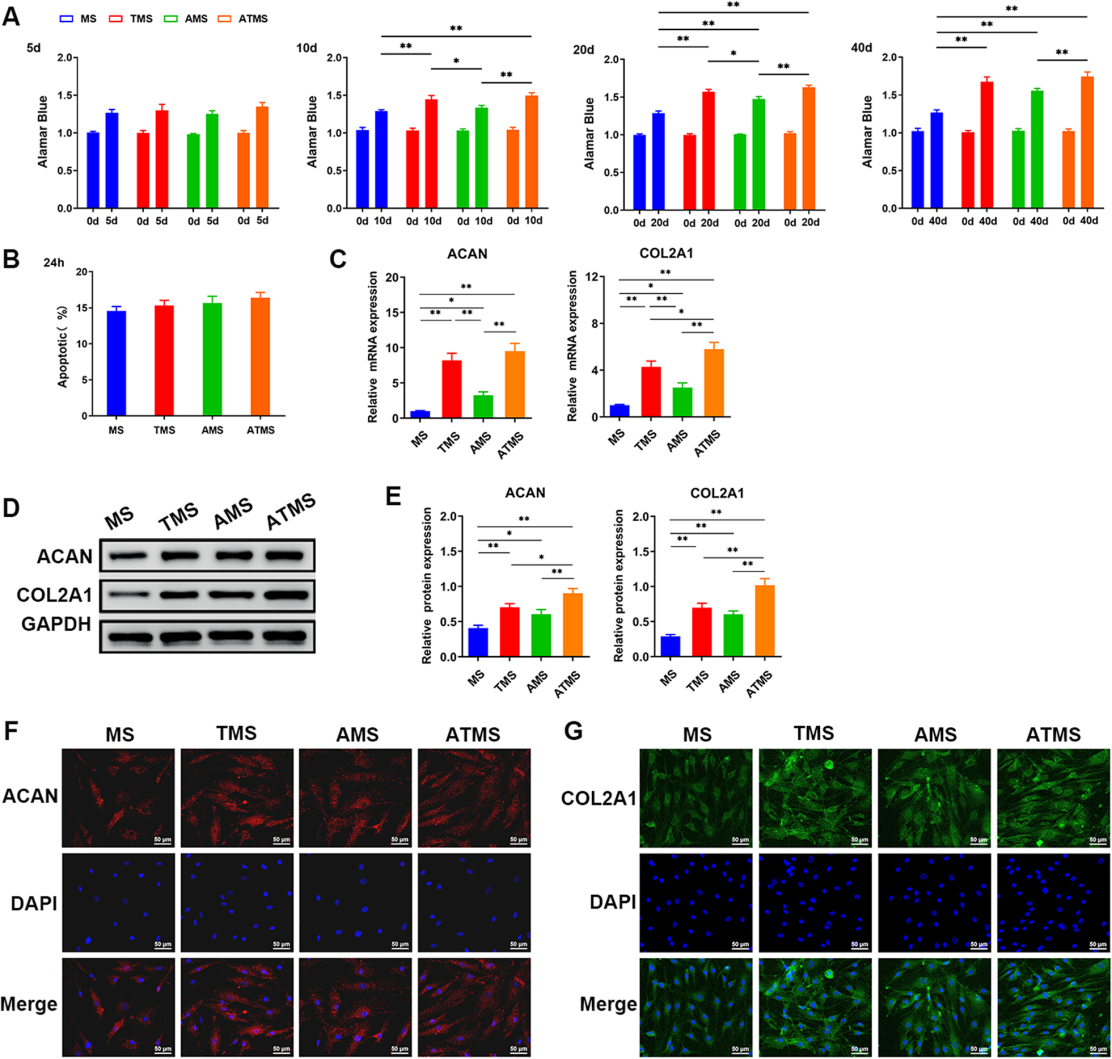
Effect of PLGA microsphere release solution on NPCs. **A** Alamarblue was used to detect the proliferation level of nucleus pulposus cells under the release of PLGA microspheres at different time points. **B** Flow cytometry was used to detect the apoptotic level of nucleus pulposus cells under the release of PLGA microspheres in different groups. **C**–**G** RT-qPCR, western blot and immunofluorescence were used to detect the expression levels of phenotypic genes and proteins of nucleus pulposus cells in different groups (*n* ≥ 3, *p < 0.05, **p < 0.01)

The effects of 5-, 10-, 20-, and 40-d release solution of PLGA microspheres on the proliferation of NPCs were observed. Compared with that in the MS group, the proliferation of NPCs was enhanced in the TMS, AMS, and ATMS groups, especially the ATMS group (Fig. 2A). Flow cytometry showed that the apoptosis did not differ between the MS, TMS, AMS, and ATMS groups (Fig. 2B). Furthermore, the gene and protein expression levels of cartilage markers (ACAN, COL2A1) were higher in the AMS, TMS, and ATMS groups (AMS < TMS < ATMS) compared with those in the MS group (Fig. 2C–G). The above results demonstrated that PLGA microspheres coloaded with TGF-β1 and anti-miR-141 can enhance the viability of NPCs and facilitate the protein-secreting capacity of the extracellular matrix of NPCs, and that combination of TGF-β1 and anti-miR-141 have the strongest effect.

### In vivo repair effect of PLGA microspheres on IDD

**Fig. 3:**
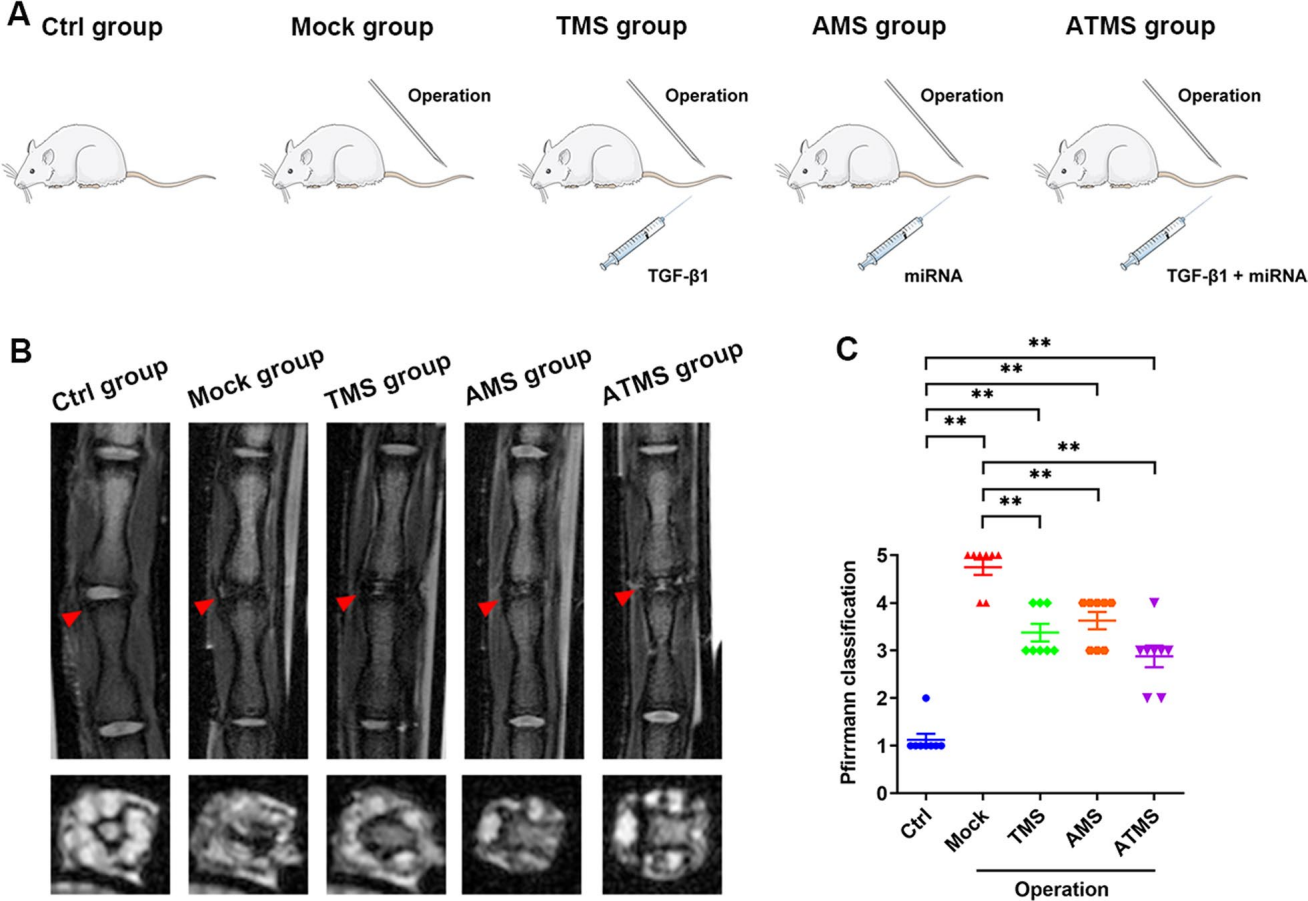
In vivo repair effect of PLGA microspheres on IDD. **A** Group description of animal experiment. **B**, **C** Observation of rat tail intervertebral disc degeneration in each group by MRI and classification analysis (*n* ≥ 3, *p < 0.05, **p < 0.01)

Whether PLGA microspheres coloaded with TGF-β1 and anti-miR-141 could promote the repair of IDD by enhancing the biological properties of NPCs was tested in the rat model of IDD established by tail puncture (Fig. 3A). MRI at 2 months after modeling showed that the intervertebral disc signal was normal in the Ctrl group, while it became significantly weakened in the Mock group due to the puncture damage, with an obvious loss of intervertebral height and the most serious degeneration among the groups. Compared with those in the Mock group, the intervertebral disc signal was only slightly decreased, with a smaller loss of intervertebral height and significantly mild degeneration, in the TMS, AMS, and ATMS groups (Fig. 3B, C).

**Fig. 4:**
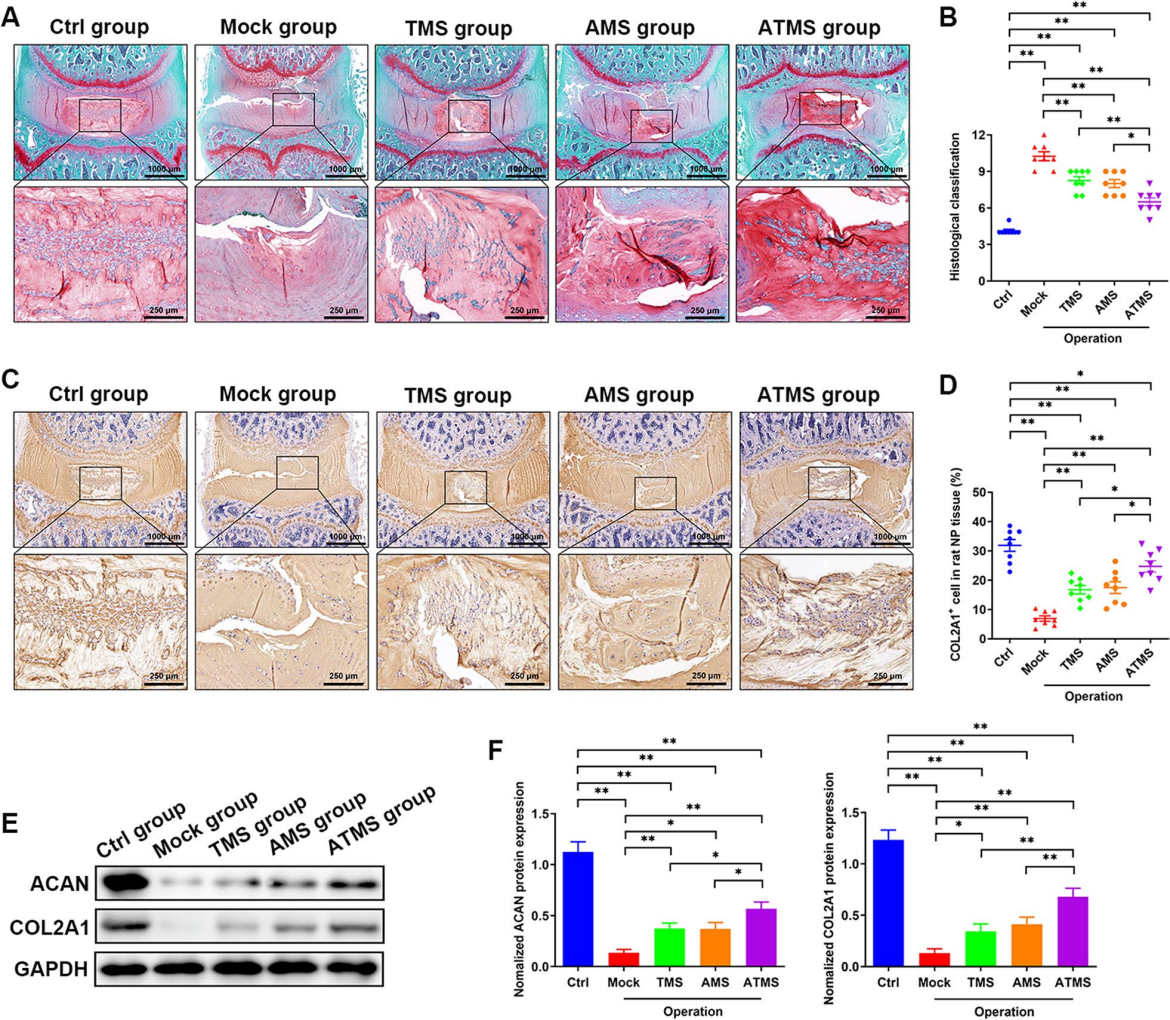
In vivo repair effect of PLGA microspheres on IDD. **A**, **B** Observation of rat tail intervertebral disc in each group by safranine and fast green staining and classification analysis. **C**, **D** The COL2A1 expression level in NP tissues in the five groups were determined by immunohistochemistry and quantitative analysis. **E**, **F** Western blot was used to detect the difference in the expression of ACAN and COL2A1 protein in nucleus pulposus tissues with different degrees of degeneration (*n* ≥ 3, *p < 0.05, **p < 0.01)

Safranin O-fast green staining showed that the NP tissues of the operative segment were evenly stained red and had an intact structure in the Ctrl group. The NP tissues of the operative segment were dehydrated and disappeared in the Mock group. In the TMS, AMS, and ATMS groups, more NPCs could still be seen, and there were still many red-stained extracellular matrix components in the operative segment, especially in the ATMS group (Fig. 4A, B). Immunohistochemistry and Western blotting showed that the levels of ACAN and COL2A1 in NPCs were significantly lower in the Mock group than in the Ctrl group, while they were significantly higher in the TMS, AMS, and ATMS groups than the Mock group, especially in the ATMS group (Fig. 4C–F).

## Discussion

Targeted delivery via microparticle systems is highly promising for the treatment of IDD [22]. First, the microparticle system can raise the chemical stability of drugs. For example, some drugs can be degraded in vivo by enzymes, such as siRNAs by RNases and proteins in the stomach by pepsin or pancreatin. This degradation can be ameliorated if the drug or the corresponding biodegradable component is loaded into a microparticle system, thereby prolonging its lifetime and increasing its efficiency. Second, the properly designed microparticle system can help improve the penetration and biodistribution of biomolecules targeting the diseased tissue. Highly biocompatible PLGA-based microspheres have been widely studied as carriers [23]. PLGA microspheres of small-molecule drugs, proteins, and plasmid DNA for in situ therapy have advantages over direct injection of drugs or gene transfection agents at the lesion site, according to previous studies on IDD [24]. Moreover, PLGA randomly aggregated by two monomers (lactic acid and glycolic acid) is a degradable functional polymer organic compound, characterized by good biocompatibility, non-toxicity, and good capsule-forming and film-forming properties [25].

The occurrence and progression of IDD result from the dysregulation of gene expression and biological factors related to various signaling pathways in NPCs. Therefore, it is necessary to promote the development of therapies combining genes and biological factors [26, 27]. Research in this field used to focus on the separate delivery of drugs or genes by different carriers, but it is difficult to deliver the biological factors and genes to the same target to exert a synergistic effect in this way. Codelivery using a properly loaded and efficiently releasing carrier has significant advantages over separate delivery [28–30]. The biological factors and genes coloaded can be simultaneously taken up by target cells, so that the two can exert a synergistic effect. Further work on novel codelivery systems is necessary for targeted treatment of IDD [31, 32].

TGF-β1 is a pleiotropic cytokine that can regulate proliferation, apoptosis, differentiation, inflammation, extracellular matrix synthesis, and developmental processes of NPCs [33, 34]. TGF-β1 is thought to reduce catabolism and inflammation by promoting matrix synthesis and inhibiting apoptosis, thereby facilitating chondrogenesis and protecting the intervertebral disc [35]. Mounting evidence has shown that miR-141 is a key player in the pathogenesis of IDD [36, 37]. Mechanistically, the NPC apoptosis induced by the crosstalk between miR-141 and SIRT1/NF-κB pathway is a key determinant of IDD, indicating that miR-141 is an important target for therapeutic intervention in IDD [38]. In this study, PLGA-based microspheres coloaded with TGF-β1 and anti-miR-141 were synthesized as carriers to repair the degenerated intervertebral disc by inhibiting NPC apoptosis and promoting NPC extracellular matrix synthesis. Finally, PLGA microspheres capable of codelivering TGF-β1 and anti-miR-141 were successfully prepared. The microspheres possessed good loading efficiency and a good release effect, so they stably and sustainably released TGF-β1 and anti-miR-141.

To test whether the microsphere release solution could enhance the biological functions of NPCs, the effects of single-loaded and coloaded microspheres on NPCs were compared between groups. Compared with that in the MS group, the release solution of single-loaded and coloaded microspheres enhanced the proliferation of NPCs as well as the extracellular secretion function to varying degrees, without inducing apoptosis. What is noteworthy is that the NPCs in the ATMS group displayed stronger proliferation capacity and extracellular secretion function than those in the TMS and AMS groups. This confirmed the synergistic effect of TGF-β1 and anti-miR-141 in vivo. Consistent results were obtained in the animal experiment: IDD was relieved better in the TMS, AMS, and ATMS groups than the Mock group, and the intervertebral disc in the ATMS group had the best MRI signal intensity and histological structure.

## Conclusion

PLGA microspheres coloaded with TGF-β1 and anti-miR-141 can effectively suppress NPC apoptosis and facilitate NPC proliferation and matrix secretion by sustainably and stably releasing prochondrogenic growth factors and therapeutic genes, thereby relieving IDD.

## Data Availability

According to the requirements, data can be obtained from the corresponding author to support the results of this study.
